# Cocirculation of 4 Dengue Virus Serotypes, Putumayo Amazon Basin, 2023–2024

**DOI:** 10.3201/eid3101.240888

**Published:** 2025-01

**Authors:** Jacob van der Ende, Victoria Nipaz, Andres Carrazco-Montalvo, Gabriel Trueba, Martin P. Grobusch, Josefina Coloma

**Affiliations:** Instituto de Microbiología, Colegio de Ciencias Biológicas y Ambientales, Universidad San Francisco de Quito, Quito, Ecuador (J. van der Ende, V. Nipaz, G. Trueba); Hospital San Miguel, Quina Care Foundation, Sucumbíos, Ecuador (J. van der Ende, M.P. Grobusch); Center for Tropical Medicine and Travel Medicine, Amsterdam UMC, Location University of Amsterdam, Amsterdam, Netherlands (J. van der Ende, M.P. Grobusch); Centro de Referencia Nacional de Genómica, Secuenciación y Bioinformática, Instituto Nacional de Investigación en Salud Pública “Leopoldo Izquieta Pérez,” Quito (A. Carrazco-Montalvo); University of California Berkeley School of Public Health, Berkeley, California, USA (J. Coloma)

**Keywords:** viruses, vector-borne infections, Dengue, DENV, Amazon, Putumayo, transmission, serotypes, Ecuador, Colombia

## Abstract

Latin America is experiencing an unprecedented dengue outbreak, causing an increased health burden. We document the cocirculation of dengue viruses 1–4 in Putumayo, a remote, underserved region at the border between Ecuador and Colombia. Dengue circulation in this largely unexplored territory represents a threat to public health in Putumayo and neighboring areas.

Latin America, including Ecuador, is in the midst of a record surge in dengue cases, causing an increased public health burden. With >1,000 deaths on the continent in 2024, the need to curb dengue virus (DENV) transmission is greater than ever before (*1*).

Until recently, little was known about which febrile illnesses are transmitted within Putumayo, an area of dense rainforest in the Amazon basin that spreads across the border between Ecuador and Colombia (*2*). In Putumayo, as well in many other regions to which malaria, other febrile illnesses, and now dengue are endemic, resources are extremely limited, and reaching a confirmed diagnosis can be challenging. Until the turn of the millennium, registered cases of acute fever in rural tropical areas of Ecuador were predominantly attributed to malaria. However, in the past 2 decades, focus has shifted to DENV as the primary etiology of febrile illness, a phenomenon observed in many parts of the world (*3*). The recent establishment of Hospital San Miguel, a secondary-level-of-care, nongovernmental organization–run hospital within Putumayo brought new resources to this area, enabling increased recognition of DENV as a cause of febrile illness.

During April–December 2023, as part of a malaria screening study (P22080M), we tested 293 community residents alongside the Putumayo River; none tested positive for malaria. To determine recent infection with DENV, we also tested symptomatic and asymptomatic persons with a rapid diagnostic test (RDT) for DENV nonstructural protein 1 (NS1), IgM, and IgG. In addition, we tested symptomatic patients experiencing fever in our hospital with the same RDT for DENV. Most samples were also tested by ELISA and quantitative reverse transcription PCR for DENV. In addition, some samples positive for DENV by quantitative reverse transcription PCR were selected for full viral genome sequencing. We derived phylogenetic trees using the maximum-likelihood method to determine the DENV genotype. This research was reviewed and approved by the ethical committee of the Universidad San Francisco de Quito: Hospital San Miguel P22080M and A2CARES 2017-0159M.

Within 1 year, we tested 204 samples collected in Putumayo for the presence of DENV with NS1/IgM/IgG RDTs, resulting in 89 samples positive for NS1, IgM, or both. Within those 89 samples, we identified all 4 serotypes of DENV (Figure 1).

**Figure 1:**
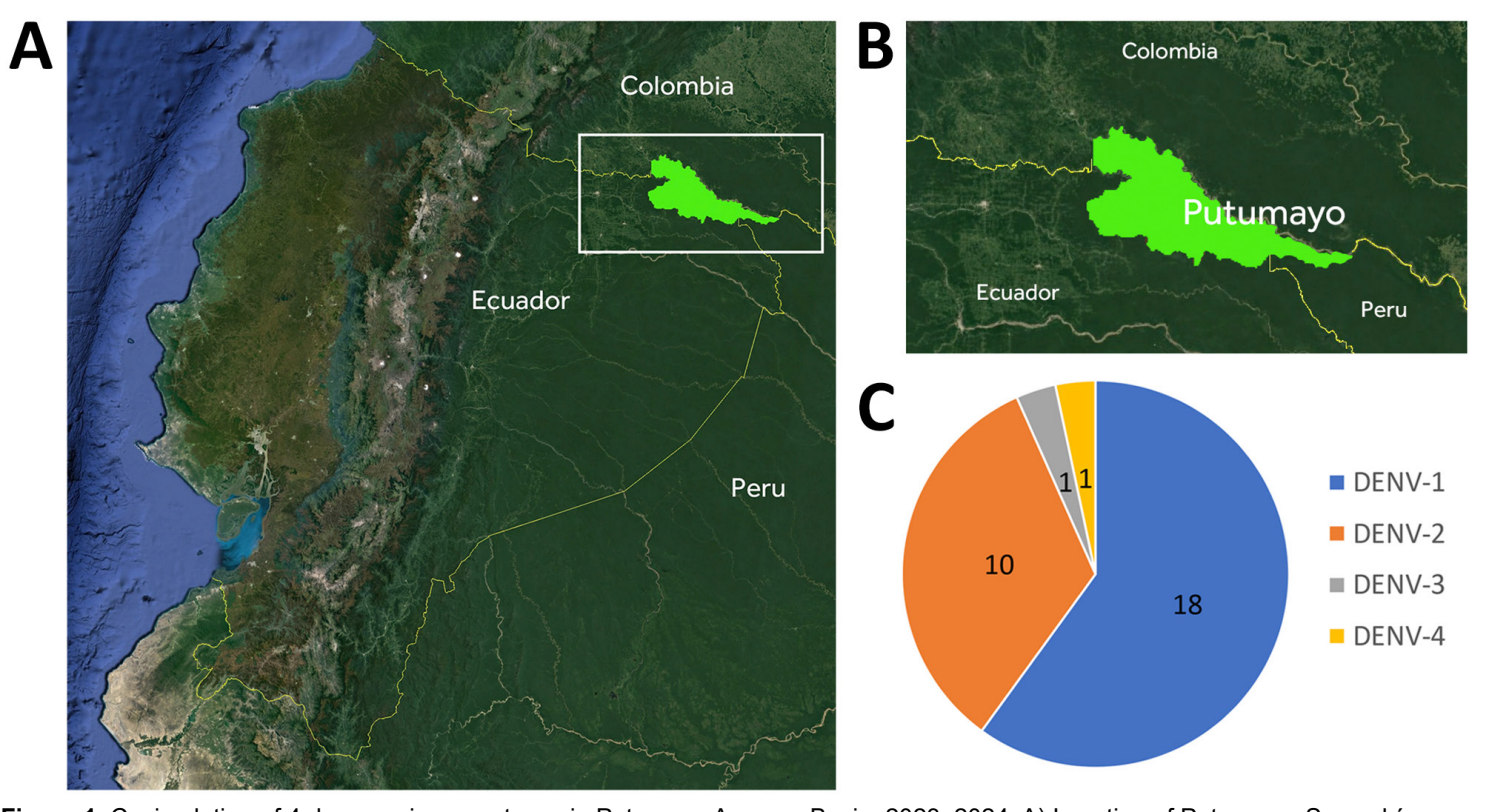
Cocirculation of 4 dengue virus serotypes in Putumayo Amazon Basin, 2023–2024. A) Location of Putumayo, Sucumbíos Province, Ecuador, South America; B) location of study area; C) representation of dengue virus serotype abundance.

Most patients for whom DENV-1 (n = 18) infection was identified were asymptomatic. Those who tested positive for DENV-2 (n = 10) were predominantly patients who sought care in our outpatient department demonstrating classical signs and symptoms of dengue. Phylogenetic analysis identified 7 DENV-1 samples as genotype V (Figure 2), which predominates in Brazil and most likely has its origins in Asia (*4*). The absence of symptoms in most patients with this genotype suggests that its transmission can go unnoticed for longer periods. Phylogenetic analysis of DENV-2 (n = 7) identified genotype III, or Southern Asian-American genotype (Figure 2), which has been evolving in the Americas for >4 decades.

**Figure 2:**
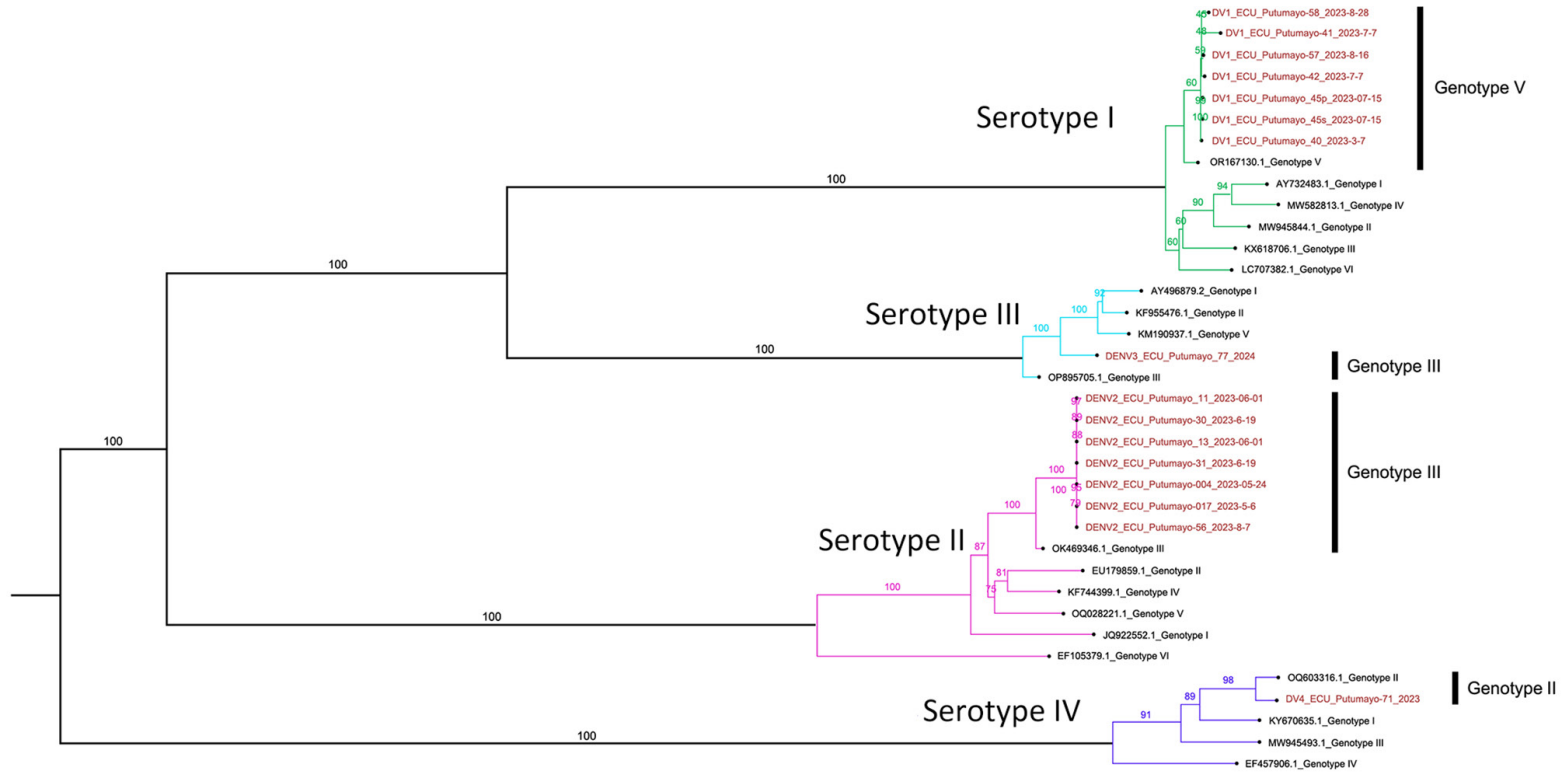
Maximum-likelihood phylogenetic tree with bootstrap values to assign specific genotypes in study of cocirculation of 4 dengue virus serotypes, Putumayo Amazon Basin, 2023–2024. Putumayo samples are shown in red.

We identified 1 case each of DENV-3 and DENV-4, both in symptomatic patients. Phylogenetic analysis of DENV-3 genotype III and of DENV-4 demonstrated genotype II (Figure 2).

Our results show that concurrent transmission of all 4 DENV serotypes is present in this largely unexplored territory with extreme environmental and human migration pressures. The high burden of dengue and different serotypes and genotypes of DENV mean that populations are more vulnerable to severity given the known immune interactions among cross-reactive responses for different serotypes (antibody enhancement of dengue) (*5*). Cross-border activities mean virus flows across countries and goes under the radar of health systems.

Unquestionably, further exploration of the dynamics and epidemiology of DENV in remote areas in Ecuador and elsewhere is of utmost importance for prompt public health responses and clinical management of severe cases. The cocirculation of 4 serotypes of DENV can represent a potentially great threat to public health within Putumayo and neighboring areas.

Appendix Additional information about cocirculation of 4 dengue virus serotypes, Putumayo Amazon Basin, 2023–2024.
